# Anticancer activity of *Zingiber ottensii* essential oil and its nanoformulations

**DOI:** 10.1371/journal.pone.0262335

**Published:** 2022-01-24

**Authors:** Pawaret Panyajai, Fah Chueahongthong, Natsima Viriyaadhammaa, Wariya Nirachonkul, Singkome Tima, Sawitree Chiampanichayakul, Songyot Anuchapreeda, Siriporn Okonogi

**Affiliations:** 1 Department of Medical Technology, Faculty of Associated Medical Sciences, Chiang Mai University, Chiang Mai, Thailand; 2 Cancer Research Unit of Associated Medical Sciences (AMS CRU), Faculty of Associated Medical Sciences, Chiang Mai University, Chiang Mai, Thailand; 3 Research Center of Pharmaceutical Nanotechnology, Chiang Mai University, Chiang Mai, Thailand; 4 Department of Pharmaceutical Science, Faculty of Pharmacy, Chiang Mai University, Chiang Mai, Thailand; Central University of Rajasthan, INDIA

## Abstract

*Zingiber ottensii*, is widely used in Asian traditional remedies for the treatment of many diseases. The present study explores anticancer activity of *Z*. *ottensii* essential oil (ZOEO) and its nanoformulations. ZOEO obtained from hydrodistillation of *Z*. *ottensii* fresh rhizomes was analysis using gas chromatography mass spectroscopy. Zerumbone (25.21%) was the major compound of ZOEO followed by sabinene (23.35%) and terpene-4-ol (15.97%). Four types of ZOEO loaded nanoformulations; nanoemulsion, microemulsion, nanoemulgels, and microemulgel, were developed. The average droplet size of the nanoemulsion and microemulsion was significantly smaller than that of the nanoemulgel and microemulgel. Comparison with other essential oils of plants of the same family on anticancer activity against A549, MCF-7, HeLa, and K562, ZOEO showed the highest cytotoxicity with IC_50_ of 43.37±6.69, 9.77±1.61, 23.25±7.73, and 60.49±9.41 μg/mL, respectively. Investigation using flow cytometry showed that ZOEO significantly increased the sub-G1 populations (cell death) in cell cycle analysis and induced cell apoptosis by apoptotic analysis. The developed nanoformulations significantly enhanced cytotoxicity of ZOEO, particularly against MCF-7 with the IC_50_ of 3.08±2.58, 0.74±0.45, 2.31±0.91, and 6.45±5.84 μg/mL, respectively. Among the four nanoformulations developed in the present study, nanoemulsion and microemulsion were superior to nanoemulgel and microemulgel in delivering ZOEO into cancer cells.

## Introduction

Cancer is a condition that involves the uncontrolled growth of cells with the ability to invade and destroy other parts of healthy tissues and organs. It is one of the diseases that threaten human death. It caused 29.8% of deaths among non-communicable diseases worldwide in 2016. It is the first or second leading cause of premature death in many countries [1]. Thus, the study of anticancer drugs has become essential for cancer patients. Nowadays, chemotherapy and radiotherapy are the conventional methods used to treat cancer patients. However, these methods can cause unpleasant side effects and toxicity since they can destroy both normal cells and cancer cells. Moreover, some patients develop resistance to therapeutic drugs during treatment, which can cause relapse.

Medicinal plants have become an interesting source of anticancer compounds because they are safe, have fewer side effects, and are easily accessible. The Zingiberaceae or ginger family is a well-known plant family in Southeast Asia. In Thailand, ginger family plants are widely used as traditional medicine for many treatments, such as to relieve stomachache and hemorrhoids, as an herbal compress for massage, to improve blood circulation, to relieve muscular pain, and as a honey balm. Moreover, the rhizomes of these plants are also used as spices and ingredients for cooking [2]. Essential oils from Zingiberaceous plants have been used for mosquito control [3, 4]. They have demonstrated various bioactivities such as antimycobacterial activity [5, 6], immunomodulatory activity [7, 8], and antineoplastic activity [9, 10].

*Zingiber ottensii* is a plant with less than 2 m in height and belongs to the Zingiberaceae family. It is widely cultured in Southeast Asian countries including Thailand, Malaysia, Indonesia, Loas, and Vietnam [11, 12]. *Z*. *ottensii* has been used as traditional medicinal herb for the treatment of various diseases in many countries. For example, in Malaysia, a poultice from leaves and rhizomes of this plant is applied to the body for postpartum care and for treatment of lumbago. In Thailand, *Z*. *ottensii* rhizomes are used to treat gastrointestinal diseases, e.g., peptic ulcers and stomachache, and constipation, as well as myalgia, sprain, bruising/contusion, and wounds. Crude extracts of *Z*. *ottensii* was reported its cytotoxicity in HEK293T/17 cells with a 50% cytotoxicity concentration (CC_50_) value of 0.266 mg/mL [13]. Furthermore, the essential oil of *Z*. *ottensii* has been found to increase cell apoptosis and reduce IL-6 levels in HeLa cells. Interestingly, it suppressed EGF-induced pAkt and pERK1/2 signaling pathway activation [14]. In addition, essential oil of *Z*. *ottensii* rhizomes (ZOEO) has been used as a topical agent for Thai traditional massage.

Nanotechnology has potential for developing anticancer drugs because of the unique properties that promote the delivery and retention of particles and enhance their permeability. Nanoparticles can be developed at a certain size for potent distribution and accumulation at cancer sites. Nanoparticles are characterized by self-assembly, stability, specificity, drug encapsulation, and compatibility as a result of their material composition. Various types of nanoparticles have been developed as drug delivery systems for medicinal purposes. Nanoemulsions and microemulsions are nanoformulations suitable for enhancing the oral bioavailability of essential oils through preparation of a homogeneous system of water, oil, surfactant, and co-surfactant [15]. Various essential oils have been used as an internal oily phase of the oil-in-water nanoemulsions and microemulsions according to the ability of these nanoformulations to improve absorption of the oils through the lipid bilayer membrane of the cells in human body. Both nanoemulsions and microemulsions can increase the bioavailability of essential oils and improve their activity at the same time. For example, nanoemulsions of ginger essential oil demonstrated an enhanced antibacterial activity against various species of bacteria [16, 17]. It has been reported that the microemulsions of essential oil from *Zingiber cassumunar* rhizome could enhance anti-inflammatory properties without cytotoxicity to normal peripheral blood mononuclear cells (PBMCs) [18]. Although *Z*. *ottensii* have demonstrated various biological activities, the anticancer activity against various cancer celles was not well reported. The present study explores the anticancer activity of ZOEO against four strains of cancer cells, A549 cells (lung carcinoma cell lines), MCF-7 cells (breast cancer cell lines), HeLa cells (cervical carcinoma cell lines), and K562 (chronic myelogenous leukemia cell line). The activity was compared with the essential oils extracted from the other three potential plants, *Alpinia galanga*, *Boesenbergia rotunda*, and *Zingiber montanum*, which are in the same family of *Z*. *ottensii*. These three plants are also used in Thai traditional medicinal remedies. Four types of ZOEO loaded nanoformulations were formulated. The anticancer activity of the formulated nanoformulations was investigated.

## Materials and methods

### Essential oil extraction and compound analysis

*Z*. *ottensii*, and 3 other plants in the same family (*A*. *galanga*, *B*. *rotunda*, *and Z*. *montanum*) were collected from a local farm located in Chiang Mai, Thailand during June 2019. All plant samples were authenticated and voucher specimens (reference no. 000109 for *Z*. *ottensii*, 009245 for *A*. *galanga*, 009724 for *B*. *rotunda*, and 004581 for *Z*. *montanum*) were deposited in the Herbarium of the Faculty of Pharmacy, Chiang Mai University, Thailand. The essential oils of the fresh rhizomes of these plants were extracted by hydrodistillation according to the method described previously [19]. The oil compositions were analyzed by gas chromatography mass spectroscopy (GC-MS) using an Agilent 6890 gas chromatography device (Agilent Technologies, Santa Clara, CA, USA) coupled to an electron impact (EI; 70 eV) HP 5973 mass selective detector (Hewlett Packard,Palo Alto, CA, USA) fitted with a column (Hewlett Packard, Palo Alto, CA, USA). The condition used for this analysis was according to that previously described [20].

### Preparation of ZOEO loaded nanoformulation

Four types of oil-in-water nanoformulations containing 10% of ZOEO were prepared. NE-ZO was prepared using 25% Tween 80 as a surfactant. The mixture composed of ZOEO, surfactant, and water was subjected to Ultra-Turrax T25 (Janke and Kunkel GmbH, Staufen, Germany) at a high-speed stirring of 12,000 rpm for 30 s to obtain to obtain a pre-emulsion. This pre-emulsion was then subjected to a high-pressure homogenizer (Micron LAB40, Homogenizer Systems, Germany) for 3 cycles to obtain a nanoemulsion. ME-ZO was prepared using a 30% surfactant mixture consisting of a 2:1 ratio of Tween 80 and cosufactant, mainly ethyl alcohol. NG-ZO and MG-ZO were prepared by adding not more than 2% of a gelling agent into NE-ZO and ME-ZO. The obtained nanoformulations were characterized for size and size distribution using a photon correlation spectrophotometer (PCS).

### Cell culture

Four strains of cancer cells, A549, MCF-7, HeLa, and K562 were used as human cancer cell models in this study. A549, MCF-7 and HeLa cells were cultured in DMEM (Dulbecco’s Modified Eagle Medium) medium (Invitrogen, Carlsbad, CA, USA) supplemented with 10% fetal bovine serum (Capricorn Scientific, Ebsdorfergrund, Germany), 100 units/mL penicillin, and 100 μg/mL streptomycin (Invitrogen, Carlsbad, CA, USA). K562 cells were cultured in RPMI (Roswell Park Memorial Institute)-1640 medium (InvitrogenTM, CA, USA) supplemented with 10% fetal bovine serum, 2 mM L-glutamine, 100 units/mL penicillin, and 100 μg/mL streptomycin (Invitrogen, Carlsbad, CA, USA). All cancer cell lines were cultured at 37°C in a humidified incubator with 5% CO_2_.

### MTT test

MTT (3-(4,5-dimethylthiazol-2-yl)-2,5-diphenyltetrazolium bromide) assay was used to detect the cytotoxicity of the test samples. A549 (5.0 × 10^3^ cells/well), MCF-7 (5.0 × 10^3^ cells/well), HeLa (5.0 × 10^3^ cells/well), and K562 (1.0 × 10^4^ cells/well) were seeded into a 96-well plate and incubated overnight at 37°C with 5% CO_2_. Then, the cells were treated with 0–100 μg/mL of the essential oils or 0–50 ng of ZOEO/mL of the nanoformulation for 48 h. After that, 15 μL of MTT dye solution (5 mg/mL) (Sigma-Aldrich, St. Louis, MO, USA) was added and incubated for 4 h. The produced formazan crystals were dissolved in 200 μL of DMSO (Sigma-Aldrich, St. Louis, MO, USA); then, the optical density was measured using an ELISA plate reader (Metertech, Taipei, Taiwan) at 578 nm with a reference wavelength of 630 nm. Four anticancer drugs, doxorubicin, idarubicin, cytarabine, and cyclophosphamide were used as positive controls. The percentage of surviving cells was calculated from the absorbance values of the test and control wells using the following equation.

Cellviability(%)=(At/Ac)×100,

where At is a mean absorbance in test well and Ac is a mean absorbance in vehicle control well. The average percentage of surviving cells at each concentration obtained from triplicate experiments was plotted as a dose-response curve. IC_50_ was defined as the lowest concentration of the test sample that inhibited cell growth by 50% compared to the untreated control.

### Trypan blue exclusion test

This test was used to confirm the cytotoxicity of the test samples. The cancer cells after exposure to the test samples under the same conditions as in the MTT test were harvested and washed with ice-cold PBS, pH 7.4 for 3 times. Then, the cells were re-suspended with PBS, pH 7.4. The cell suspensions were diluted with PBS, pH 7.4 at the appropriate dilution before being mixed with 0.2% trypan blue solution at 1:2 dilution for cell count on a hemocytometer. From this test, the viable cells and the dead cells could also be obviously detected.

### Cell cycle analysis

This experiment was used to determine the effects of ZOEO on cell cycle arrest and cell death. MCF-7 was used as a cancer cell model. The cells were treated with ZOEO at concentrations of 2, 3, and 10 μg/mL as these concentrations were the IC_20_, IC_30_, and IC_50_ of the oil, respectively. After 48 h, the cells were harvested and washed using PBS, pH 7.4 for 3 times. After washing, the cells were fixed with 70% ethanol in PBS, pH 7.4 for 30 min and incubated on ice. After that, the cells were washed again with PBS, pH 7.4 for 2 times and stained with propidium iodide (PI) solution (0.02 mg/mL PI in PBS, pH 7.4). The rates of cell death and cell cycle distribution were analyzed using flow cytometry (Cytomics FC500, Beckman Coulter, Pasadena, CA, USA).

### Apoptosis assay

To determine the effects of ZOEO on apoptotic effect in cancer cells, MCF-7 cells (5 × 10^4^ cells/mL) were treated with ZOEO at the IC_20_, IC_30_, and IC_50_ (2, 3, 10 μg/mL, respectively) for 48 h. Then, cells were stained using Biolegend^TM^ FITC Annexin V Apoptosis Detection Kit with PI according to the manufacturer’s instructions. Briefly, cells were harvested and then washed using PBS, pH 7.4 for 3 times. After washing, cells were resuspended with 100 μL of binding buffer, then, cells were stained with Annexin V-FITC and PI for 15 min in the dark at room temperature. Next, each sample was added with 400 μL of binding buffer. The percentages of cell population were analyzed using flow cytometry (Cytomics FC500, Beckman Coulter, Pasadena, CA, USA).

### Statistical analysis

All experiments were performed in triplicate. The average of triplicate experiments and standard derivation (SD) were used for quantification. The levels of cell populations were compared to the vehicle control in each experiment. The results are shown as mean ± S.D. The SPSS statistics software ver. 22 (SPSS Inc., USA) was used for statistical analysis. Differences between the means of each sample were analyzed by one-way analysis of variance (one-way ANOVA), followed by LSD post-hoc analysis. Statistical significance was considered at p < 0.05 and p < 0.001.

The average of triplicate experiments and standard derivation (SD) were used for quantification. The levels of cell populations were compared to the vehicle control in each experiment. The results are shown as mean ± S.D. Differences between the means of each sample were analyzed by one-way analysis of variance (one-way ANOVA). Statistical significance was considered at p < 0.05 and p < 0.001.

### Results and discussion

#### Yield and chemical analysis of ZOEO

After subjecting to hydrodistillation for 3 h, the fresh rhizomes of *Z*. *ottensii* yielded 0.21±0.12% oil, almost same as that of the other plants in the same family as shown in Table 1. The outer appearance of the obtained ZOEO was a pale yellowish oil with a distinctive camphorous odor.

**Table 1:** Percent yield of essential oils from Zingiberaceae family.

Plant essential oil	Yield (%)
*A*. *galanga*	0.21 ± 0.09
*B*. *rotunda*	0.11 ± 0.08
*Z*. *montanum*	0.68 ± 0.07
*Z*. *ottensii*	0.21 ± 0 .12

Data represent mean ± standard deviation (SD).

Chemical constituents of the extracted ZOEO analyzed by GC-MS demonstrated 18 identified compounds representing 98.02% of the total oil including mainly zerumbone (25.21%), sabinene (23.35%), and terpene-4-ol (15.67%) as shown in Table 2 and S1 Fig. *Z*. *ottensii* can be found in many countries in Southeast Asia, including Thailand, Indonesia, Malaysia, Laos, Myanmar, and Vietnam. The essential oil compositions of this plant have been reported from several countries, e.g. Malaysia [21, 22], Thailand [23, 24], and Indonesia [25]. Compared to the previous studies, the compositions of ZOEO obtained from the present study was slightly different. In general, the composition profile, concentration of individual components, and yield of the essential oils of one plant can be different depending on intrinsic factors such as plant cultivars [26–28] and extrinsic factors, e.g., plant cultivars, environmental factors, cultivation conditions, geographical location, and harvest time [29–34].

**Table 2:** Main compounds of ZOEO by GC-MS.

Retention time (min)	Compound	Relative concentration (%)
5.64	α-thujene	0.53
5.83	α-pinene	2.52
7.16	sabinene	23.35
7.23	β-pinene	8.31
7.84	β-myrcene	0.71
8.75	bornylene	0.49
9.08	thymene	3.94
9.21	dl-limonene	0.90
9.32	eucalyptol	2.03
10.46	1,8-cineol	1.53
10.94	γ-terpentene	0.34
15.67	terpene-4-ol	15.97
16.37	α-terpineol	0.55
25.88	trans-caryophyllene	0.39
27.29	α-humulene	9.67
33.04	α-caryophyllene	0.86
33.41	humulene oxide	0.71
37.97	zerumbone	25.21

### Preparation of ZOEO loaded nanoformulation

Research in nanotechnology is increasing rapidly. Nanotechnology can be applied in medical and pharmaceutical fields to make advances in diagnosis and treatment. Anticipated applications include *in vivo* and *in vitro* drug delivery [35, 36], drug solubility and stability enhancement [37, 38], and production of improved biocompatible materials [39]. Several types of nanoformulations have been formulated and showed their advantages [40]. In this study, four types of ZOEO loaded nanoformulations, nanoemulsion (NE-ZO), microemulsion (ME-ZO), nanoemulgel (NG-ZO), and microemulgel (MG-ZO) have been successfully prepared. Particle characterization using PCS revealed that all of the ZOEO droplets in the obtained nanoformulations were within the nanoscale range as shown in Table 3. Moreover, the size distribution expressed as polydispersity index (PDI) of the nanoparticles obtained in each formulation was in the accepted range (0.1–0.3) for pharmaceutical use. The zeta potential of the NE-ZO and ME-ZO were near zero because of the non-ionic surfactant (Tween 80) used in the systems. It was considered that slightly higher negative value of NG-ZO and MG-ZO than NE-ZO and ME-ZO was according to the effect of a gelling agent used in the systems.

**Table 3:** Size, size distribution, and zetapotential of ZOEO loaded nanoformulations.

Nanoformulations	Size (nm)	PDI	Zeta potential (mV)
NE-ZO	13.8 ± 0.2	0.152 ± 0.045	-4.44 ± 0.92
ME-ZO	21.2 ± 0.2	0.267 ± 0.013	-7.57 ± 0.32
NG-ZO	99.5 ± 2.7	0.320 ± 0.007	-19.67 ± 0.96
MG-ZO	99.2 ± 4.4	0.358 ± 0.026	-23.33 ± 2.38

Data represent mean ± standard deviation (SD).

### Cytotoxicity of ZOEO by MTT test

The results show that each plant oil possessed different cytotoxic effects against the test cancer cells as the different IC_50_ was obtained as shown in Table 4. ZOEO showed significantly high anticancer activity against the four tested cancer cells and demonstrated the highest cytotoxicity against MCF-7 with IC_50_ of 9.77 ± 1.61 μg/mL. *A*. *galanga*, *B*. *rotunda*, and *Z*. *montanum* are the plants in the Zingiberaceae family and used in the Asian traditional remedies for treatment of various diseases related to cancer. The results obviously showed that ZOEO possessed significantly higher cytotoxic effects against all four tested cells than the essential oils of the other three plants. Moreover, ZO essential oil was previously reported the strong decreasing of the cell viability in HeLa cells when the concentration was increased by MTT assay [14]. The results also showed that among the four cancer cells, only MCF-7 and HeLa could be inhibited by the essential oils of these three plants. The essential oils of *A*. *galanga* and *B*. *rotunda* had no cytotoxic effect on A549 and A562 cells. In addition, *Z*. *montanum* oil could not inhibit A549. Comparing with the anticancer drugs used as positive control, ZOEO showed higher effectively anticancer activity than cyclophosphamide against all test cells and cytarabine against A549 and K562.

**Table 4:** Cytotoxicity of the essential oils and drugs against four cancer cells by MTT test.

Plant Essential oil or drug	IC_50_			
	A549	MCF-7	HeLa	K562
*A*. *galanga* (μg/mL)	> 100	93.00 ± 5.33	82.09 ± 7.46	> 100
*B*. *rotunda* (μg/mL)	> 100	19.61 ± 2.26	30.39 ± 9.64	> 100
*Z*. *montanum* (μg/mL)	> 100	70.55 ± 4.92	68.80 ± 2.98	93.58 ± 4.59
*Z*. *ottensii* (μg/mL)	43.37 ± 6.69	9.77 ± 1.61	23.25 ± 7.73	60.49 ± 9.41
Doxorubicin (ng/mL)	293.59 ± 10.48	15.82 ± 4.97	26.08 ± 1.79	800.45 ± 60.06
Idarubicin (ng/mL)	37.44 ± 9.42	5.58 ± 2.98	4.16 ± 1.42	409.40 ± 38.48
Cytarabine (μg/mL)	> 100	1.87 ± 0.34	18.11 ± 3.45	> 100
Cyclophosphamide (μg/mL)	> 400	> 400	> 400	> 400

Data represent mean ± standard deviation (SD). The dataset is available in S1−S4 Tables.

### Effects of ZOEO on total cell number and cell cycle analysis

**Fig 1:**
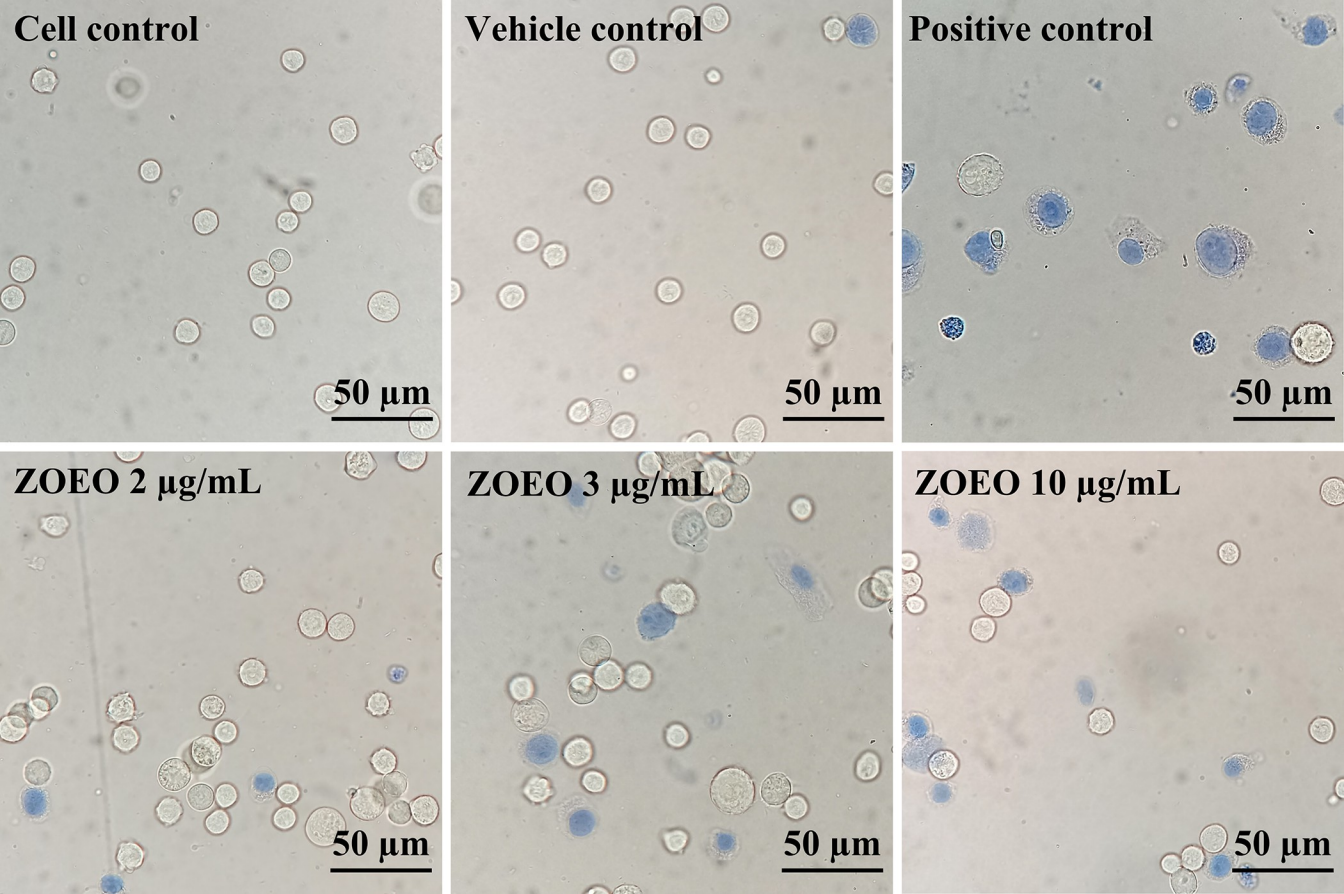
Images (40×) of MCF-7 cells after contacted with various concentrations of ZOEO and the controls. Viable cells show colorless and dead cells show blue.

**Fig 2:**
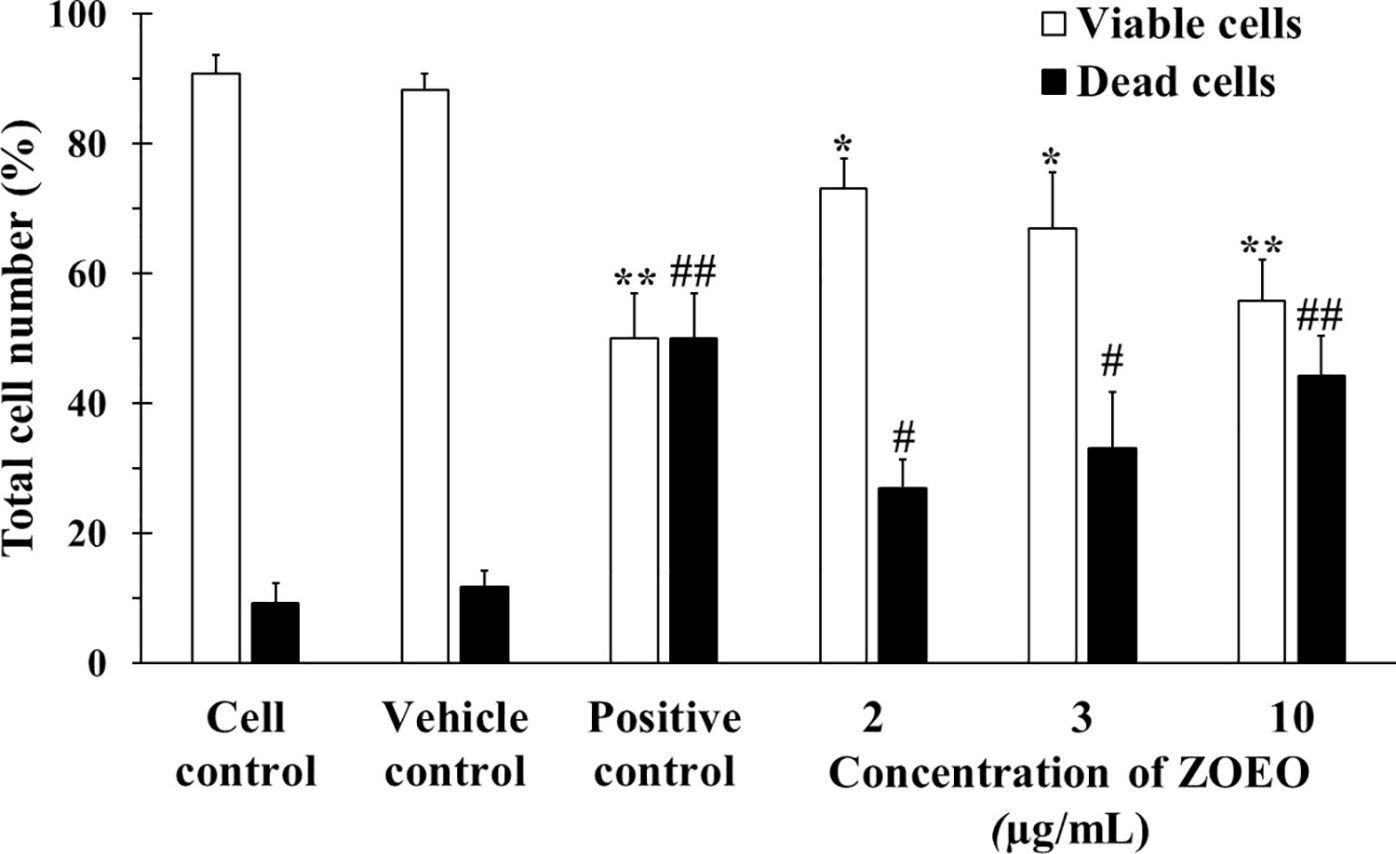
Total number of viable and dead MCF-7 cells after 48 h in cell control (CC), vehicle control (VC), and various concentrations of ZOEO, in comparison with the positive control (cytarabine at 2 μg/mL). Each bar represents mean ± SD of three independent experiments performed in triplicate. Asterisk (*) denotes significant differences from viable cells in vehicle control; ** p < 0.001. Sharp (#) denotes significant differences from dead cells in vehicle control; ## p < 0.001. The dataset is available in S5 Table.

Cytotoxicity to cancer cells of ZOEO was confirmed using trypan blue exclusion assay and flow cytometry. MCF-7 was used as a cancer cell model and cytarabine (2 μg/mL) was used as a positive control due to no autofluorescence. Trypan blue is a dye that can penetrate cell membrane and stain the cytoplasm of the cells. The viable cells were not stained and showed a clear cytoplasm because they had ability to exclude trypan blue whereas the dead cells showed the blue cytoplasm as they could not repulse the dye as shown in Fig 1. From this result, relatively large number of blue cells can be observed in the images of the cells after exposed to the positive control and high concentrations of ZOEO. After exposed to the lowest ZOEO concentration (2 μg/mL), the image shows a similar number of blue cells that were contacted with the cell control and the vehicle control groups. To confirm the number of dead and viable cells, the cells were counted using a hemocytometer. The results are illustrated in Fig 2. It is obviously seen that apoptosis increased as the concentration of ZOEO increased.

**Fig 3:**
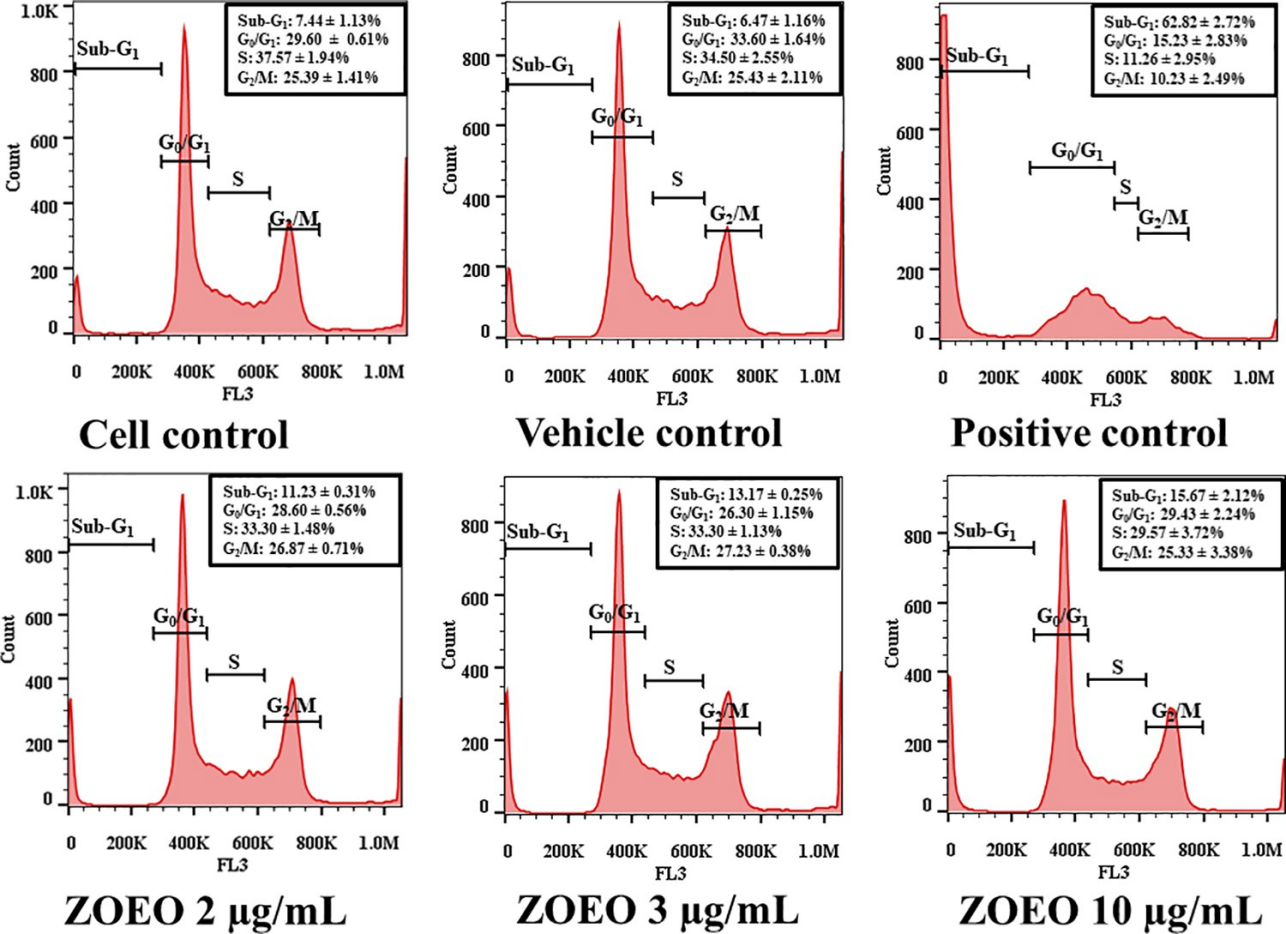
Cell cycle distribution of MCF-7 after 48 h exposed to various concentrations of ZOEO in comparison with the controls. Sub sub-G1 peaks are indicated by red arrows. The dataset is available in S6 Table.

**Fig 4:**
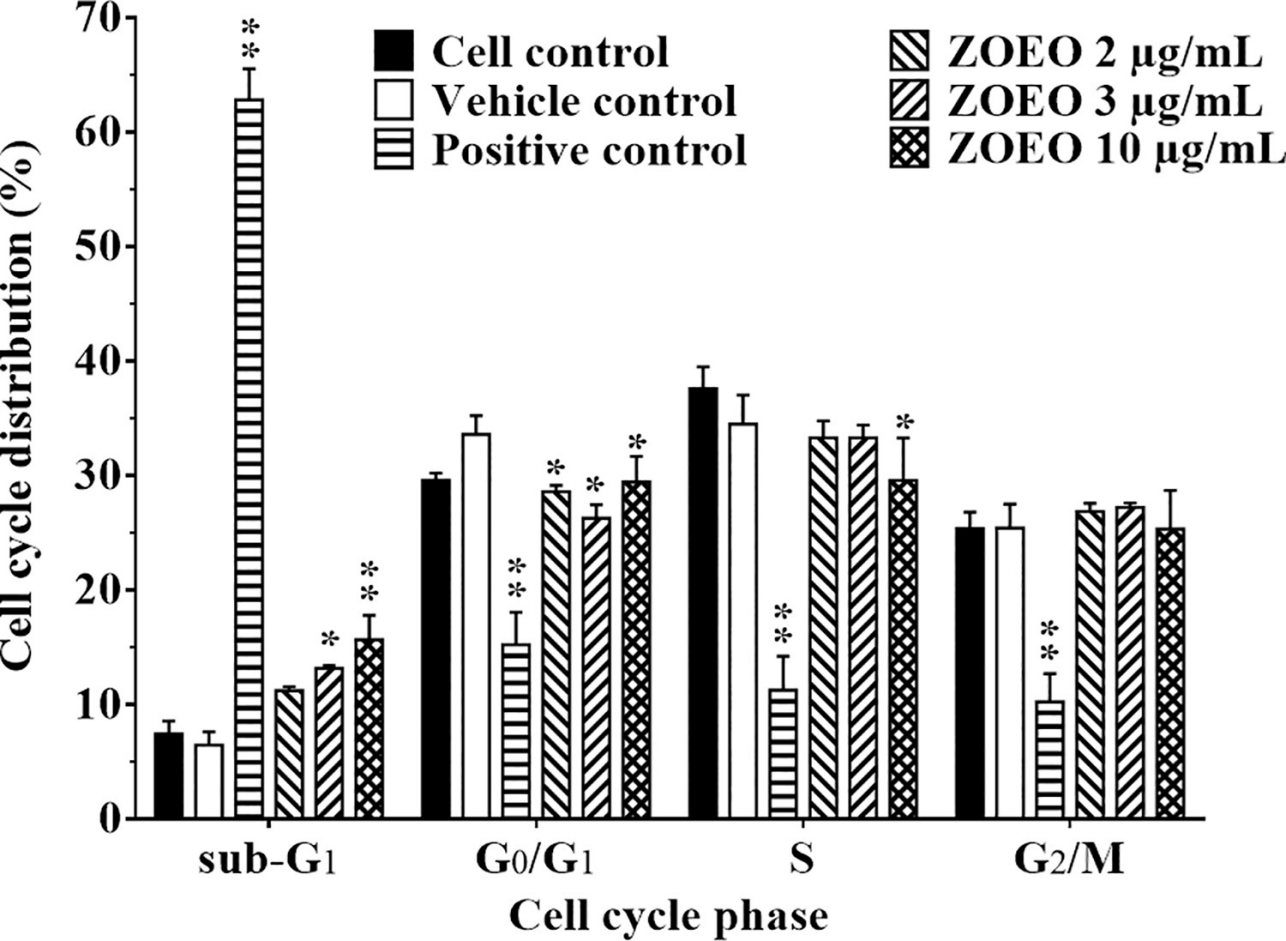
Representative bar graph of cell cycle phases of MCF-7 cells after 48 h exposed to various concentrations of ZOEO in comparison with the controls. Each bar represents mean ± SD of three independent experiments performed in triplicate. Asterisk (*) denotes significant differences from vehicle control; * p < 0.05; ** p < 0.001. The dataset is available in S6 Table.

Anticancer activity of ZOEO was deeply investigated using flow cytometry. Cell cycle analysis at sub-G1 population represents dead cells was observed. The result as demonstrated in Fig 3 shows different size of sub-G1 peaks. After analyzing cell populations in each phase of cell cycle, the result demonstrates in Fig 4. Comparison to the cell control and the vehicle control, it is clearly confirmed that ZOEO possesses anticancer activity. In addition, after 48 h exposure to ZOEO at 2, 3, and 10 μg/mL, the sub-G1 cell population increased respectively. The obtained results confirmed that the anticancer activity of ZOEO is dose dependent.

### Effect of ZOEO on cell apoptosis

**Fig 5:**
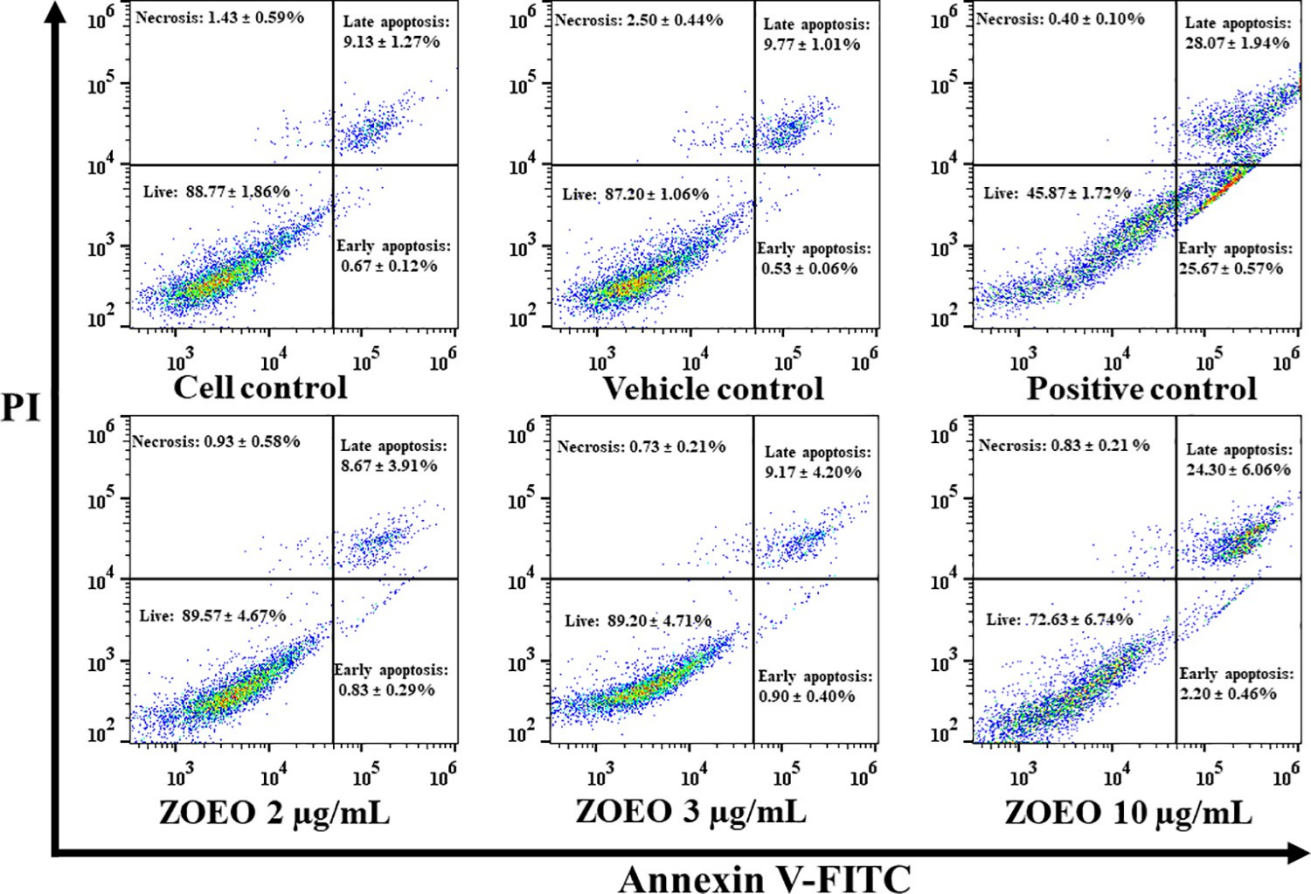
Apoptosis assay by flow cytometry after staining with double annexin V-FITC/propidium iodide (PI). MCF-7 cells were treated with ZOEO at the concentrations of 2, 3, and 10 μg/mL. Representative flow cytometry dot plot indicating the cell population in apoptotic and necrotic quadrants after treatment with various concentrations. The dataset is available in S7 Table.

**Fig 6:**
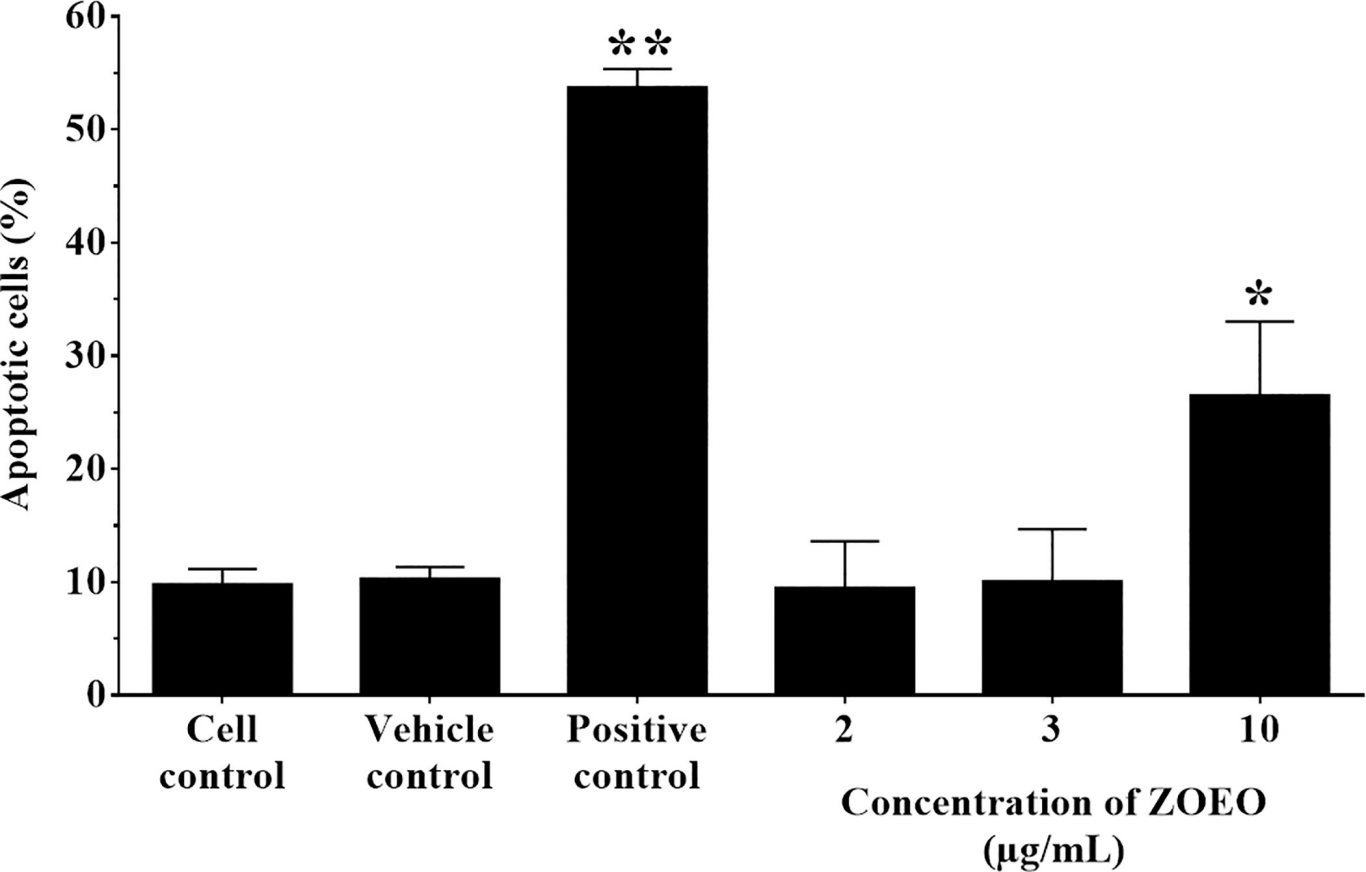
Representative bar graph of apoptotic cells (%) by flow cytometer after ZOEO treatments in various concentrations. MCF-7 cells were treated with ZOEO at the concentrations of 2, 3, and 10 μg/mL and stained with double annexin V-FITC/propidium iodide (PI). The percentage of apoptotic cells was statistically compared. Each bar represents mean ± SD of three independent experiments performed in triplicate. Asterisk (*) denotes significant differences from vehicle control; * p < 0.05, ** p < 0.001. The dataset is available in S7 Table.

This study was performed to confirm the effect of ZOEO on cell apoptosis that showed at the sub-G1 phase of cell cycle analysis. The percentage of apoptotic cells were measured after treatment with ZOEO at the concentration of 2, 3, and 10 μg/mL. Cytarabine (2 μg/mL) was used as a positive control (53.73 ± 1.63%). As shown in Figs 5 and 6, ZOEO at a concentration of 10 μg/mL significantly increased the fraction of apoptotic cells (26.50 ± 6.52%, P < 0.05) compared to cell control and vehicle control (9.80 ± 1.35% and 10.30 ± 1.05%, respectively). This result related to the ZO essential oil on HeLa cells. It induced apoptotic HeLa cells approximately 10–20% in HeLa cells treated with 1:6,000 and 1:3,000 dilutions [14]. ZOEO at the concentrations of 2 and 3 μg/mL showed the % apoptotic cells with values of 9.50 ± 4.12 and 10.07 ± 4.59%, respectively. There was no significant difference when compared to those of cell control and vehicle control. However, ZOEO showed a trend to increase cell apoptosis by a dose dependent manner. This result indicated that ZOEO improved the ability to induce apoptosis.

### Cytotoxicity of ZOEO loaded nanoformulations

Subjecting to the MTT assay for investigating anticancer activity against four cancer cells, A549, MCF-7, HeLa, and K562, the results as shown in Table 5 demonstrated that the formulated nanofomulations possessed significantly stronger cytotoxicity than ZOEO alone, whereas the blank formulations had no activity. Each ZOEO loaded nanoformulation showed different cytotoxic effects on the tested cancer cells. Among them, NE-ZO and ME-ZO demonstrated stronger anticancer activity than NG-ZO and MG-ZO, respectively. Gel bases are usually prepared from various kinds of polymers as gelling agents. Adding nanoemulsion or microemulsion with suitable gelling agent can yield nanoemulgels or microemulgel, respectively. The obtained emulgel nanoformulations are suitable for topical drug delivery, however, the formulations prolong drug release [41]. The retardation of drug release depends on the type of gelling agent [42]. It is considered that higher anticancer activity of NE-ZO and ME-ZO than NG-ZO and MG-ZO is due to the retardation effects on ZOEO release of the gel matrix. Among the cancer cells, MCF-7 exhibited the most sensitive to ZOEO and the formulated ZOEO nanoformulations. The IC_50_ of ZOEO alone (9.77 ± 1.61 μg/mL) was significantly higher than that of NE-ZO and ME-ZO (1.08 ± 2.58 and 0.74 ± 0.45 ng/mL, respectively), indicating the significantly higher efficiency of these nanoformulations to deliver ZOEO into the cancer cells.

**Table 5:** Cytotoxicity of ZOEO loaded nanoformulations against four cancer cells.

Nanoformulations	IC_50_ value (ng of essential oil/mL)			
	A549	MCF-7	HeLa	K562
NE-ZO	18.45 ± 3.33	1.08 ± 2.58	5.81 ± 2.38	32.48 ± 1.21
NE-ZO blank	> 50	> 50	> 50	> 50
ME-ZO	28.24 ± 12.51	0.74 ± 0.45	7.24 ± 2.49	33.31 ± 2.37
ME-ZO blank	> 50	> 50	> 50	> 50
NG-ZO	33.76 ± 8.26	4.31 ± 0.91	8.88 ± 1.97	35.35 ± 1.72
NG-ZO blank	> 50	> 50	> 50	> 50
MG-ZO	36.74 ± 4.31	6.45 ± 5.84	11.01 ± 2.54	36.23 ± 2.48
MG-ZO blank	> 50	> 50	> 50	> 50

Data represent mean ± standard deviation (SD). The dataset is available in S8−S11 Tables.

## Conclusion

In the current work, the anticancer activity of ZO-EO on four cancer cells, namely A549, MCF-7, HeLa, and K562, was investigated and compared with essential oils from three other plants in the same family. ZO-EO possesses cytotoxic effects on all four cancer cells significantly higher than the essential oils of the other three plants. Among the four cancer cells, ZO-EO shows the most effective in inhibiting MCF-7. The anticancer activity of ZO-EO is dose dependent that induces cell death. This effect can be confirmed using flow cytometry where the sub-G1 cell population increases in cell cycle analysis and apoptotic induction by apoptosis assay after exposure with ZO-EO at 2, 3, and 10 μg/mL. To improve ZO-EO efficacy, it was produced in a nanoparticle form. Loading ZO-EO in the nanoformulations can significantly enhance anticancer effect of ZO-EO. Among the formulated nanoformulations, NE-ZO and ME-ZO show better anticancer activity than NG-ZO and MG-ZO. Both ZO-EO and the nanoformulations demonstrated the specific cytotoxicity in breast cancer when compared to other cancer cell types. Our findings support the potential of ZO-EO and nanoformulations (NE-ZO and ME-ZO) as an anticancer, especially in patients with breast cancer.

## Supporting information

S1 Fig GC chromatogram of ZOEO. (TIF)

S1 Table Cytotoxicity of the essential oils and drugs against A549 cells by MTT test. (PDF)

S2 Table Cytotoxicity of the essential oils and drugs against MCF-7 cells by MTT test. (PDF)

S3 Table Cytotoxicity of the essential oils and drugs against HeLa cells by MTT test. (PDF)

S4 Table Cytotoxicity of the essential oils and drugs against K562 cells by MTT test. (PDF)

S5 Table Total numbers of MCF-7 cells following cytarabine or ZOEO treatment were compared with cell control. (PDF)

S6 Table Cell cycle distribution of MCF-7 after 48 h exposed to various concentrations of ZOEO in comparison with the controls. (PDF)

S7 Table Apoptosis assay by flow cytometry after staining with double annexin V-FITC/propidium iodide (PI). (PDF)

S8 Table Cytotoxicity of ZOEO loaded nanoformulations against A549 cells. (PDF)

S9 Table Cytotoxicity of ZOEO loaded nanoformulations against MCF-7 cells. (PDF)

S10 Table Cytotoxicity of ZOEO loaded nanoformulations against HeLa cells. (PDF)

S11 Table Cytotoxicity of ZOEO loaded nanoformulations against K562 cells. (PDF)

## References

[1] Cao B, Soerjomataram I, Bray F. The burden and prevention of premature deaths from noncommunicable diseases, including cancer: a global perspective. World Cancer Rep Cancer Res Cancer Prev Lyon, Fr Int Agency Res Cancer. 2020.

[2] Viriyaadhammaa N, Saiai A, Neimkhum W, Nirachonkul W, Chaiyana W, Chiampanichayakul S, et al. Cytotoxic and Antiproliferative Effects of Diarylheptanoids Isolated from Curcuma comosa Rhizomes on Leukaemic Cells. Molecules. 2020/11/27. 2020;25. doi: 10.3390/molecules25225476 33238470 PMC7700379

[3] Rajeswary M, Govindarajan M, Alharbi NS, Kadaikunnan S, Khaled JM, Benelli G. Zingiber cernuum (Zingiberaceae) essential oil as effective larvicide and oviposition deterrent on six mosquito vectors, with little non-target toxicity on four aquatic mosquito predators. Environ Sci Pollut Res Int. 2017/05/13. 2018;25: 10307–10316. doi: 10.1007/s11356-017-9093-3 28497331

[4] Govindarajan M, Rajeswary M, Arivoli S, Tennyson S, Benelli G. Larvicidal and repellent potential of Zingiber nimmonii (J. Graham) Dalzell (Zingiberaceae) essential oil: an eco-friendly tool against malaria, dengue, and lymphatic filariasis mosquito vectors? Parasitol Res. 2016;115: 1807–1816. doi: 10.1007/s00436-016-4920-x 26792432

[5] Baldin VP, Bertin de Lima Scodro R, Mariano Fernandez CM, Ieque AL, Caleffi-Ferracioli KR, Dias Siqueira VL, et al. Ginger essential oil and fractions against Mycobacterium spp. J Ethnopharmacol. 2019/07/22. 2019;244: 112095. doi: 10.1016/j.jep.2019.112095 31325601

[6] Chen IN, Chang CC, Ng CC, Wang CY, Shyu YT, Chang TL. Antioxidant and antimicrobial activity of Zingiberaceae plants in Taiwan. Plant Foods Hum Nutr. 2008;63: 15–20. doi: 10.1007/s11130-007-0063-7 18157743

[7] Alambra JR, Alenton RRR, Gulpeo PCR, Mecenas CL, Miranda AP, Thomas RC, et al. Immunomodulatory effects of turmeric, Curcuma longa (Magnoliophyta, Zingiberaceae) on Macrobrachium rosenbergii (Crustacea, Palaemonidae) against Vibrio alginolyticus (Proteobacteria, Vibrionaceae). AACL Bioflux. 2012;5: 13–17.

[8] Carrasco FR, Schmidt G, Romero AL, Sartoretto JL, Caparroz-Assef SM, Bersani-Amado CA, et al. Immunomodulatory activity of Zingiber officinale Roscoe, Salvia officinalis L. and Syzygium aromaticum L. essential oils: evidence for humor- and cell-mediated responses. J Pharm Pharmacol. 2009/07/11. 2009;61: 961–967. doi: 10.1211/jpp/61.07.0017 19589240

[9] Lima DAN, Pelegrini BB, Uechi FAA, Varago RC, Pimenta BB, Kaneshima AMS, et al. Evaluation of Antineoplasic Activity of Zingiber Officinale Essential Oil in the Colorectal Region of Wistar Rats. Asian Pacific J Cancer Prev. 2020/07/28. 2020;21: 2141–2147. doi: 10.31557/APJCP.2020.21.7.2141 32711443 PMC7573421

[10] Santos P, Avanço GB, Nerilo SB, Marcelino RIA, Janeiro V, Valadares MC, et al. Assessment of cytotoxic activity of rosemary (Rosmarinus officinalis L.), turmeric (Curcuma longa L.), and ginger (Zingiber officinale R.) essential oils in cervical cancer cells (HeLa). Sci World J. 2016;2016. doi: 10.1155/2016/9273078 28042599 PMC5155122

[11] Huong LT, Ly SN, Truong VB. Zingiber ottensii Valeton (Zingiberaceae) ― a newly recorded species for Vietnam. Biosci Discov. 2016;7: 93–96.

[12] Aung M, Tanaka N. Seven Taxa of Zingiber (Zingiberaceae) Newly Recorded for the Flora of Myanmar. Bull Natl Museum Nat Sci Ser B, Bot. 2019;45: 1–8.

[13] Khammaneejan O, Aratsittisin J, Roytrakul S, Nimlamool W, Okonogi S, Wikan N, et al. Anti-proliferative Activity of Zingiberaceae Crude Extracts against Human Embryonic Kidney Cell Line (HEK 293T/17). RSU Int Res Conf. 2020;1: 404–411.

[14] Ruttanapattanakul J, Wikan N, Chinda K, Jearanaikulvanich T, Krisanuruks N, Muangcha M, et al. Essential oil from zingiber ottensii induces human cervical cancer cell apoptosis and inhibits mapk and pi3k/akt signaling cascades. Plants. 2021;10. doi: 10.3390/plants10071419 34371622 PMC8309419

[15] Kale SN, Deore SL. Emulsion micro emulsion and nano emulsion: a review. Syst Rev Pharm. 2017;8: 39.

[16] Mostafa NM. Antibacterial activity of ginger (Zingiber officinale) leaves essential oil nanoemulsion against the cariogenic Streptococcus mutans. J Appl Pharm Sci. 2018;8: 34–41.

[17] Firoozi M, Rezapour‐Jahani S, Shahvegharasl Z, Anarjan N. Ginger essential oil nanoemulsions: Preparation and physicochemical characterization and antibacterial activities evaluation. J Food Process Eng. 2020;43: e13434.

[18] Chaiyana W, Anuchapreeda S, Leelapornpisid P, Phongpradist R, Viernstein H, Mueller M. Development of Microemulsion Delivery System of Essential Oil from Zingiber cassumunar Roxb. Rhizome for Improvement of Stability and Anti-Inflammatory Activity. AAPS PharmSciTech. 2016/08/10. 2017;18: 1332–1342. doi: 10.1208/s12249-016-0603-2 27502407

[19] Okonogi S, Prakatthagomol W, Ampasavate C, Klayraung S. Killing kinetics and bactericidal mechanism of action of Alpinia galanga on food borne bacteria. African J Microbiol Res. 2011. 10.5582/ddt.2011.v5.2.84 22466145

[20] Okonogi S, Chaiyana W. Enhancement of anti-cholinesterase activity of Zingiber cassumunar essential oil using a microemulsion technique. Drug Discov Ther. 2012. doi: 10.5582/ddt.2012.v6.5.249 23229145

[21] Sirat HM, Nordin AB. Essential oil of Zingiber ottensii Valeton. J Essent Oil Res. 1994;6: 635–636. doi: 10.1080/10412905.1994.9699356

[22] Malek SNA, Ibrahim H, Lai HS, Serm LG, Seng CK, Yusoff MM, et al. Essential oils of Zingiber ottensii Vealet. and Zingiber zerumbet (L.) Sm. Malasian J Sci. 2005;24: 49–58.

[23] Thubthimthed S, Limsiriwong P, Rerk-Am U, Suntorntanasat T. Chemical composition and cytotoxic activity of the essential oil of Zingiber ottensii. Acta Hortic. 2005;675: 107–109. doi: 10.17660/ActaHortic.2005.675.14

[24] Theanphong O. Chemotaxonomic study of volatile oils from rhizomes of 9 Zingiber species (Zingiberaceae). Thai J Bot. 2016;8: 127–139.

[25] Marliani L, Subarnas A, Moelyono MW, Halimah E, Pratiwi FW, Suhardiman A. Essential Oil components of leaves and rhizome of zingiber ottensii val. from Bandung, Indonesia. Res J Chem Environ. 2018;22: 54–57.

[26] Patel RP, Singh R, Rao BRR, Singh RR, Srivastava A, Lal RK. Differential response of genotype × environment on phenology, essential oil yield and quality of natural aroma chemicals of five Ocimum species. Ind Crops Prod. 2016;87: 210–217. doi: 10.1016/j.indcrop.2016.04.001

[27] Liber Z, Carović-Stanko K, Politeo O, Strikić F, Kolak I, Milos M, et al. Chemical characterization and genetic relationships among ocimum basilicum L. cultivars. Chem Biodivers. 2011;8: 1978–1989. doi: 10.1002/cbdv.201100039 22083911

[28] Sadeghi H, Robati Z, Saharkhiz MJ. Variability in Zataria multiflora Bioss. essential oil of twelve populations from Fars province, Iran. Ind Crops Prod. 2015;67: 221–226. doi: 10.1016/j.indcrop.2015.01.021

[29] Vidic D, Maksimović M, Ćaavar S, Siljak-Yakovlev S. Influence of the continental climatic conditions on the essential-oil composition of Salvia brachyodon Vandas transferred from adriatic coast. Chem Biodivers. 2010;7: 1208–1216. doi: 10.1002/cbdv.200900126 20491077

[30] Sirousmehr A, Arbabi J, Asgharipour MR. Effect of drought stress levels and organic manures on yield, essential oil content and some morphological characteristics of sweet basil (Ocimumbasilicum). Adv Environ Biol. 2014;8: 1322–1327.

[31] Singh M, Guleria N. Influence of harvesting stage and inorganic and organic fertilizers on yield and oil composition of rosemary (Rosmarinus officinalis L.) in a semi-arid tropical climate. Ind Crops Prod. 2013;42: 37–40. doi: 10.1016/j.indcrop.2012.04.054

[32] Gazim ZC, Ana Carolina LA, Hovell AMC, Rezende CM, Nascimento IA, Ferreira GA, et al. Seasonal variation, chemical composition, and analgesic and antimicrobial activities of the essential oil from leaves of Tetradenia riparia (Hochst.) Cdd in suthern Brazil. Molecules. 2010;15: 5509–5524. doi: 10.3390/molecules15085509 20714310 PMC6257709

[33] Djouahri A, Boualem S, Boudarene L, Baaliouamer A. Geographic’s variation impact on chemical composition, antioxidant and anti-inflammatory activities of essential oils from wood and leaves of Tetraclinis articulata (Vahl) Masters. Ind Crops Prod. 2015;63: 138–146. doi: 10.1016/j.indcrop.2014.10.018

[34] Formisano C, Delfine S, Oliviero F, Tenore GC, Rigano D, Senatore F. Correlation among environmental factors, chemical composition and antioxidative properties of essential oil and extracts of chamomile (Matricaria chamomilla L.) collected in Molise (South-central Italy). Ind Crops Prod. 2015;63: 256–263. doi: 10.1016/j.indcrop.2014.09.042

[35] De Jong WH, Borm PJA. Drug delivery and nanoparticles: Applications and hazards. Int J Nanomedicine. 2008;3: 133–149. doi: 10.2147/ijn.s596 18686775 PMC2527668

[36] Ferrari M. Cancer nanotechnology: Opportunities and challenges. Nat Rev Cancer. 2005;5: 161–171. doi: 10.1038/nrc1566 15738981

[37] Anantaworasakul P, Okonogi S. Encapsulation of Sesbania grandiflora extract in polymeric micelles to enhance its solubility, stability, and antibacterial activity. J Microencapsul. 2017;34: 73–81. doi: 10.1080/02652048.2017.1284277 28097930

[38] Naksuriya O, van Steenbergen MJ, Torano JS, Okonogi S, Hennink WE. A Kinetic Degradation Study of Curcumin in Its Free Form and Loaded in Polymeric Micelles. AAPS J. 2016;18: 777–787. doi: 10.1208/s12248-015-9863-0 27038456 PMC5256596

[39] Naksuriya O, Shi Y, Van Nostrum CF, Anuchapreeda S, Hennink WE, Okonogi S. HPMA-based polymeric micelles for curcumin solubilization and inhibition of cancer cell growth. Eur J Pharm Biopharm. 2015;94: 501–512. doi: 10.1016/j.ejpb.2015.06.010 26134273

[40] Jeevanandam J, Chan YS, Danquah MK. Nano-formulations of drugs: Recent developments, impact and challenges. Biochimie. 2016;128–129: 99–112. doi: 10.1016/j.biochi.2016.09.021 27436182

[41] Ashara KC, Paun JS, Soniwala MM, Chavada JR, Mori NM. ChemInform Abstract: Micro-Emulsion Based Emulgel: A Novel Topical Drug Delivery System. ChemInform. 2014;45: no-no. doi: 10.1002/chin.201434272

[42] Saeio K, Pongpaibul Y, Viernstein H, Okonogi S. Factors influencing drug dissolution characteristics from hydrophilic ploymer. Sci Pharm. 2007;163: 147–163.

