# Supplementary figures and images for: Anticancer activity of Zingiber ottensii essential oil and its nanoformulations
Source: PLoS One. 2022 Jan 24;17(1):e0262335. doi: 10.1371/journal.pone.0262335 (PMC8786151; doi:10.1371/journal.pone.0262335)

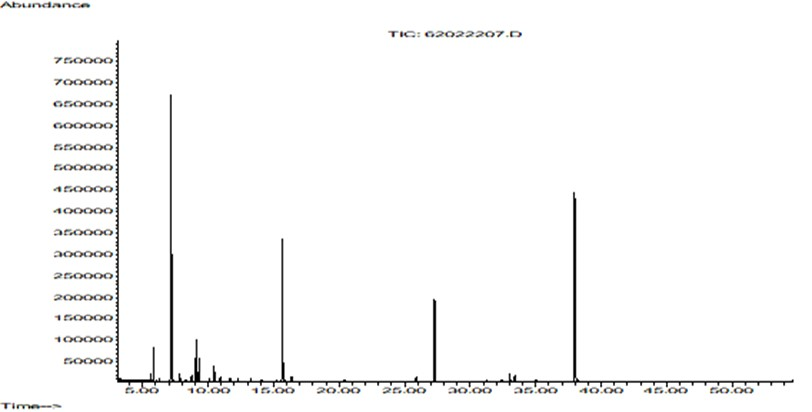

Supplement: S1 Fig — (TIF) [file pone.0262335.s001.tif]
