# Supplementary material for: Anticancer activity of Zingiber ottensii essential oil and its nanoformulations
Source: PLoS One. 2022 Jan 24;17(1):e0262335. doi: 10.1371/journal.pone.0262335 (PMC8786151; doi:10.1371/journal.pone.0262335)
Supplement: S1 Table — (PDF) [file pone.0262335.s002.pdf]

S1 Table. Cytotoxicity of the essential oils and drugs against A549 cells by MTT test.

**Plant essential oil or drug**	**IC_{50} value**				
	1	2	3	Mean	SD
A. galanga (µg/mL)	>100	>100	>100	>100	-
B. rotunda (µg/mL)	>100	>100	>100	>100	-
C. aeruginosa (µg/mL)	24.20	29.51	35.25	29.66	5.53
C. longa (µg/mL)	38.18	52.37	50.72	47.09	7.76
C. mangga (µg/mL)	71.29	73.40	69.53	71.41	1.93
Z. montanum (µg/mL)	>100	>100	>100	>100	-
Z. officinale (µg/mL)	33.93	35.97	35.25	35.05	1.03
Z. ottensii (µg/mL)	38.74	49.14	51.23	46.37	6.69
Doxorubicin (ng/mL)	283.25	304.20	293.31	293.59	10.48
Idarubicin (ng/mL)	26.65	44.01	41.65	37.44	9.42
Cytarabine (µg/mL)	>100	>100	>100	>100	-
Cyclophosphamide (µg/mL)	>400	>400	>400	>400	-
