# Supplementary material for: Anticancer activity of Zingiber ottensii essential oil and its nanoformulations
Source: PLoS One. 2022 Jan 24;17(1):e0262335. doi: 10.1371/journal.pone.0262335 (PMC8786151; doi:10.1371/journal.pone.0262335)
Supplement: S2 Table — (PDF) [file pone.0262335.s003.pdf]

S2 Table. Cytotoxicity of the essential oils and drugs against MCF-7 cells by MTT test.

**Plant essential oil or drug**	**IC_{50} value**				
	1	2	3	Mean	SD
A. galanga (µg/mL)	96.99	86.95	95.07	93.00	5.33
B. rotunda (µg/mL)	17.49	21.99	19.35	19.61	2.26
C. aeruginosa (µg/mL)	18.80	20.89	20.85	20.18	1.20
C. longa (µg/mL)	36.49	39.84	44.75	40.36	4.15
C. mangga (µg/mL)	34.75	32.94	34.34	34.01	0.95
Z. montanum (µg/mL)	72.87	64.90	73.89	70.55	4.92
Z. officinale (µg/mL)	24.83	19.93	29.15	24.64	4.61
Z. ottensii (µg/mL)	9.26	11.57	8.48	9.77	1.61
Doxorubicin (ng/mL)	11.96	10.08	18.85	13.63	4.62
Idarubicin (ng/mL)	8.09	2.29	6.38	5.58	2.98
Cytarabine (µg/mL)	2.23	1.56	1.83	1.87	0.34
Cyclophosphamide (µg/mL)	>400	>400	>400	>400	-
