# Supplementary material for: Anticancer activity of Zingiber ottensii essential oil and its nanoformulations
Source: PLoS One. 2022 Jan 24;17(1):e0262335. doi: 10.1371/journal.pone.0262335 (PMC8786151; doi:10.1371/journal.pone.0262335)
Supplement: S3 Table — (PDF) [file pone.0262335.s004.pdf]

S3 Table. Cytotoxicity of the essential oils and drugs against HeLa cells by MTT test.

**Essential oil or drug**	**IC_{50} value**				
	1	2	3	Mean	SD
A. galanga (µg/mL)	106.27	95.33	81.35	94.32	12.49
B. rotunda (µg/mL)	24.04	41.51	24.62	30.06	9.92
C. aeruginosa (µg/mL)	17.89	15.59	17.49	16.99	1.23
C. longa (µg/mL)	33.07	23.34	23.67	26.69	5.52
C. mangga (µg/mL)	45.57	42.04	36.82	41.48	4.40
Z. montanum (µg/mL)	92.73	79.12	101.08	90.97	11.08
Z. officinale (µg/mL)	17.13	17.67	27.81	20.87	6.01
Z. ottensii (µg/mL)	22.43	31.35	15.97	23.25	7.73
Doxorubicin (ng/mL)	24.19	17.38	26.32	22.63	4.67
Idarubicin (ng/mL)	3.46	5.78	3.22	4.16	1.42
Cytarabine (µg/mL)	2.18	1.76	1.49	1.81	0.35
Cyclophosphamide (µg/mL)	>400	>400	>400	>400	-
