# Supplementary material for: Anticancer activity of Zingiber ottensii essential oil and its nanoformulations
Source: PLoS One. 2022 Jan 24;17(1):e0262335. doi: 10.1371/journal.pone.0262335 (PMC8786151; doi:10.1371/journal.pone.0262335)
Supplement: S4 Table — (PDF) [file pone.0262335.s005.pdf]

S4 Table. Cytotoxicity of the essential oils and drugs against K562 cells by MTT test.

**Plant essential oil or drug**	**IC_{50} value**				
	1	2	3	Mean	SD
A. galanga (µg/mL)	>100	>100	>100	>100	-
B. rotunda (µg/mL)	>100	>100	>100	>100	-
C. aeruginosa (µg/mL)	12.18	14.13	13.99	13.43	1.09
C. longa (µg/mL)	19.90	24.98	27.34	24.07	3.80
C. mangga (µg/mL)	29.76	34.01	30.66	31.48	2.23
Z. montanum (µg/mL)	96.66	88.31	95.77	93.58	4.59
Z. officinale (µg/mL)	18.85	16.00	17.33	17.39	1.43
Z. ottensii (µg/mL)	59.70	70.27	51.49	60.49	9.41
Doxorubicin (ng/mL)	752.88	780.53	867.94	800.45	60.06
Idarubicin (ng/mL)	374.60	402.88	450.71	409.40	38.48
Cytarabine (µg/mL)	>100	>100	>100	>100	-
Cyclophosphamide (µg/mL)	>400	>400	>400	>400	-
