# Supplementary material for: Anticancer activity of Zingiber ottensii essential oil and its nanoformulations
Source: PLoS One. 2022 Jan 24;17(1):e0262335. doi: 10.1371/journal.pone.0262335 (PMC8786151; doi:10.1371/journal.pone.0262335)
Supplement: S5 Table — (PDF) [file pone.0262335.s006.pdf]

S5 Table. Total numbers of MCF-7 cells following cytarabine or ZOEO treatment were compared with cell control.

Treatments	Viable cells (%)					Dead cells (%)				
	1	2	3	Mean	SD	1	2	3	Mean	SD
Cell control	93.33	91.25	87.50	90.69	2.96	6.67	8.75	12.50	9.31	2.96
Vehicle control	87.64	91.10	86.21	88.31	2.52	12.36	8.90	13.79	11.69	2.52
Positive control	57.41	49.18	43.48	50.02	7.00	42.59	50.82	56.52	49.98	7.00
ZOEO 2 μg/mL	77.50	73.43	68.45	73.13	4.53	22.50	26.57	31.55	26.87	4.53
ZOEO 3 μg/mL	74.91	68.29	57.72	66.97	8.67	25.09	31.71	42.28	33.03	8.67
ZOEO 10 μg/mL	62.12	55.67	49.69	55.83	6.22	37.88	44.33	50.31	44.17	6.22
