# Supplementary material for: Anticancer activity of Zingiber ottensii essential oil and its nanoformulations
Source: PLoS One. 2022 Jan 24;17(1):e0262335. doi: 10.1371/journal.pone.0262335 (PMC8786151; doi:10.1371/journal.pone.0262335)
Supplement: S6 Table — (PDF) [file pone.0262335.s007.pdf]

S6 Table. Cell cycle distribution of MCF-7 after 48 h exposed to various concentrations of ZOEO in comparison with the controls.

Treatments	Sub G_1 population (%)				
	1	2	3	Mean	SD
Cell control	6.6	7	8.73	7.44	1.13
Vehicle control	6.3	7.7	5.4	6.47	1.16
Positive control	65.27	59.9	63.3	62.82	2.72
ZOEO 2 µg/mL	11.5	10.9	11.3	11.23	0.31
ZOEO 3 µg/mL	12.9	13.2	13.4	13.17	0.25
ZOEO 10 µg/mL	13.3	16.3	17.4	15.67	2.12

Treatments	G_0/G_1 population (%)				
	1	2	3	Mean	SD
Cell control	29.9	30	28.9	29.60	0.61
Vehicle control	34	31.8	35	33.60	1.64
Positive control	11.99	17.2	16.5	15.23	2.83
ZOEO 2 µg/mL	28.7	28	29.1	28.60	0.56
ZOEO 3 µg/mL	25.1	27.4	26.4	26.30	1.15
ZOEO 10 µg/mL	32	28.4	27.9	29.43	2.24

Treatments	S population (%)				
	1	2	3	Mean	SD
Cell control	39.7	37.1	35.9	37.57	1.94
Vehicle control	32.3	37.3	33.9	34.50	2.55
Positive control	13.97	11.7	8.12	11.26	2.95
ZOEO 2 µg/mL	32.3	35	32.6	33.30	1.48
ZOEO 3 µg/mL	34.6	32.6	32.7	33.30	1.13
ZOEO 10 µg/mL	26.5	33.7	28.5	29.57	3.72

Treatments	G_2/M population (%)				
	1	2	3	Mean	SD
Cell control	23.8	25.9	26.47	25.39	1.41
Vehicle control	27.4	23.2	25.7	25.43	2.11
Positive control	7.40	11.2	12.08	10.23	2.49
ZOEO 2 µg/mL	27.5	26.1	27	26.87	0.71
ZOEO 3 µg/mL	27.4	26.8	27.5	27.23	0.38
ZOEO 10 µg/mL	28.2	21.6	26.2	25.33	3.38
