# Supplementary material for: Anticancer activity of Zingiber ottensii essential oil and its nanoformulations
Source: PLoS One. 2022 Jan 24;17(1):e0262335. doi: 10.1371/journal.pone.0262335 (PMC8786151; doi:10.1371/journal.pone.0262335)
Supplement: S7 Table — (PDF) [file pone.0262335.s008.pdf]

S7 Table. Apoptosis assay by flow cytometry after staining with double annexin V-FITC/propidium iodide (PI).

**Treatments**	**Apoptotic cells (%)**				
	1	2	3	Mean	SD
Cell control	8.3	10.2	10.9	9.80	1.35
Vehicle control	10.4	11.3	9.2	10.30	1.05
Positive control	52.3	53.4	55.5	53.73	1.63
ZOEO 2 μg/mL	5.9	8.6	14	9.50	4.12
ZOEO 3 μg/mL	6.1	9	15.1	10.07	4.59
ZOEO 10 μg/mL	32	19.3	28.2	26.50	6.52

S8. Cytotoxicity of ZOEO loaded nanoformulations against A549 cells (Table 5).

Nanoformulations	IC_{50} value (ng of essential oil/mL)				
	1	2	3	Mean	SD
NE-ZO-S	19.87	20.82	14.64	18.45	3.33
NE-ZO-B	>50	>50	>50	>50	-
ME-ZO-S	25.21	17.53	41.97	28.24	12.50
ME-ZO-B	>50	>50	>50	>50	-
NG-ZO-S	35.03	29.49	45.74	36.76	8.26
NG-ZO-B	>50	>50	>50	>50	-
MG-ZO-S	32.52	30.16	38.53	33.74	4.31
MG-ZO-B	>50	>50	>50	>50	-

S9. Cytotoxicity of ZOEO loaded nanoformulations against MCF-7 cells (Table 5).

Nanoformulations	IC_{50} value (ng of essential oil/mL)				
	1	2	3	Mean	SD
NE-ZO-S	5.97	1.00	2.28	3.08	2.58
NE-ZO-B	>50	>50	>50	>50	-
ME-ZO-S	1.22	0.65	0.34	0.74	0.45
ME-ZO-B	>50	>50	>50	>50	-
NG-ZO-S	2.25	3.24	1.41	2.30	0.91
NG-ZO-B	>50	>50	>50	>50	-
MG-ZO-S	3.77	13.14	2.44	6.45	5.84
MG-ZO-B	>50	>50	>50	>50	-

S10. Cytotoxicity of ZOEO loaded nanoformulations against HeLa cells (Table 5).

**Nanoformulations**	**IC_{50} value (ng of essential oil/mL)**				
	1	2	3	Mean	SD
NE-ZO-S	4.16	8.54	4.74	5.81	2.38
NE-ZO-B	>50	>50	>50	>50	-
ME-ZO-S	4.67	9.65	7.41	7.24	2.49
ME-ZO-B	>50	>50	>50	>50	-
NG-ZO-S	8.49	7.14	11.01	8.88	1.97
NG-ZO-B	>50	>50	>50	>50	-
MG-ZO-S	8.75	10.52	13.75	11.01	2.54
MG-ZO-B	>50	>50	>50	>50	-

S11. Cytotoxicity of ZOEO loaded nanoformulations against K562 cells (Table 5).

**Nanoformulations**	**IC_{50} value (ng of essential oil/mL)**				
	1	2	3	Mean	SD
NE-ZO-S	31.18	33.57	32.70	32.48	1.21
NE-ZO-B	>50	>50	>50	>50	-
ME-ZO-S	30.61	34.26	35.06	33.31	2.37
ME-ZO-B	>50	>50	>50	>50	-
NG-ZO-S	34.18	37.33	34.54	35.35	1.72
NG-ZO-B	>50	>50	>50	>50	-
MG-ZO-S	30.36	34.69	34.63	33.23	2.48
MG-ZO-B	>50	>50	>50	>50	-

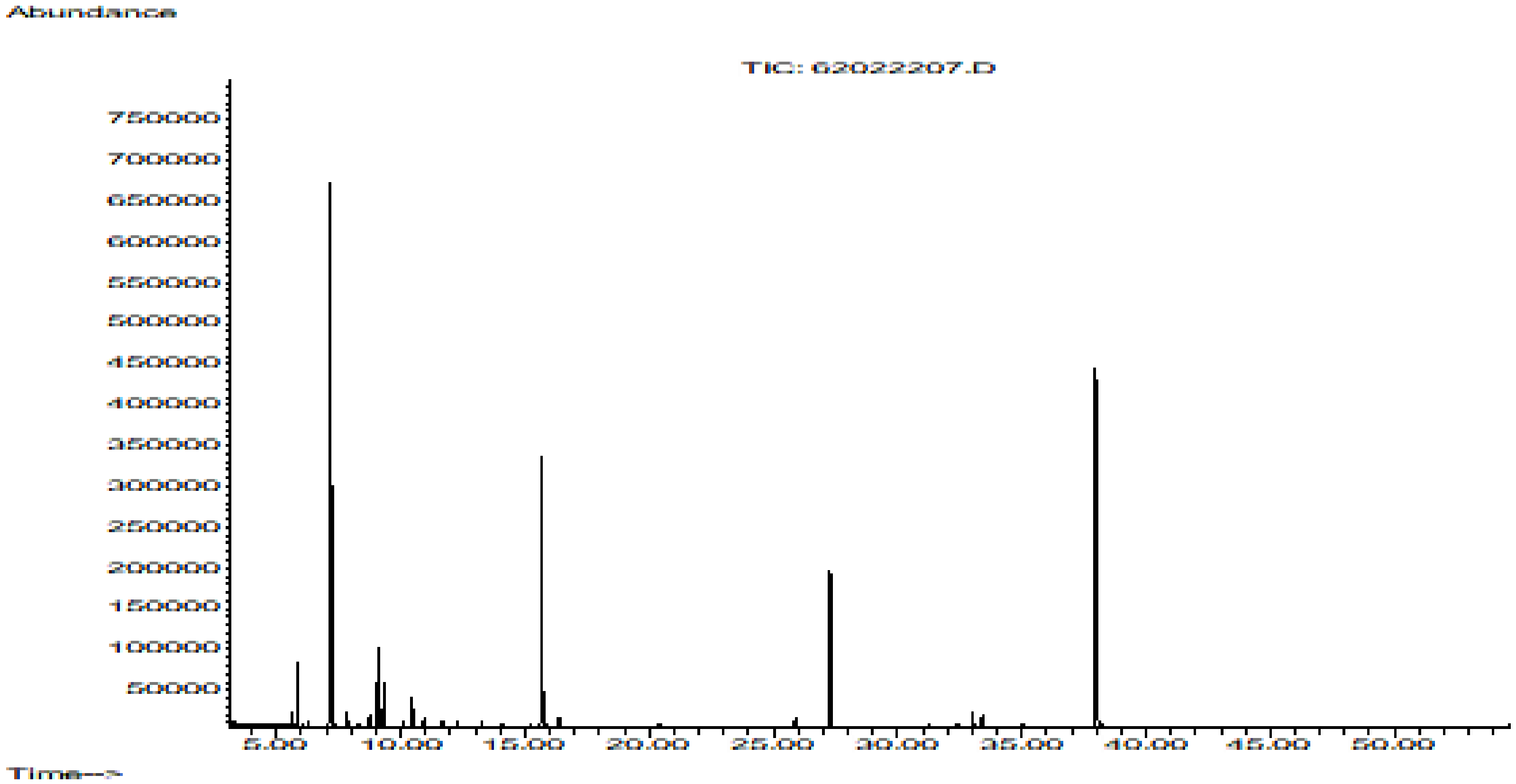

S12. GC chromatogram of ZOEO.
