# Supplementary material for: Anticancer activity of Zingiber ottensii essential oil and its nanoformulations
Source: PLoS One. 2022 Jan 24;17(1):e0262335. doi: 10.1371/journal.pone.0262335 (PMC8786151; doi:10.1371/journal.pone.0262335)
Supplement: S8 Table — (PDF) [file pone.0262335.s009.pdf]

S8 Table. Cytotoxicity of ZOEO loaded nanoformulations against A549 cells.

Nanoformulations	IC_{50} value (ng of essential oil/mL)				
	1	2	3	Mean	SD
NE-ZO-S	19.87	20.82	14.64	18.45	3.33
NE-ZO-B	>50	>50	>50	>50	-
ME-ZO-S	25.21	17.53	41.97	28.24	12.50
ME-ZO-B	>50	>50	>50	>50	-
NG-ZO-S	35.03	29.49	45.74	36.76	8.26
NG-ZO-B	>50	>50	>50	>50	-
MG-ZO-S	32.52	30.16	38.53	33.74	4.31
MG-ZO-B	>50	>50	>50	>50	-
