# Supplementary material for: Anticancer activity of Zingiber ottensii essential oil and its nanoformulations
Source: PLoS One. 2022 Jan 24;17(1):e0262335. doi: 10.1371/journal.pone.0262335 (PMC8786151; doi:10.1371/journal.pone.0262335)
Supplement: S9 Table — (PDF) [file pone.0262335.s010.pdf]

S9 Table. Cytotoxicity of ZOEO loaded nanoformulations against MCF-7 cells.

Nanoformulations	IC_{50} value (ng of essential oil/mL)				
	1	2	3	Mean	SD
NE-ZO-S	5.97	1.00	2.28	3.08	2.58
NE-ZO-B	>50	>50	>50	>50	-
ME-ZO-S	1.22	0.65	0.34	0.74	0.45
ME-ZO-B	>50	>50	>50	>50	-
NG-ZO-S	2.25	3.24	1.41	2.30	0.91
NG-ZO-B	>50	>50	>50	>50	-
MG-ZO-S	3.77	13.14	2.44	6.45	5.84
MG-ZO-B	>50	>50	>50	>50	-
