# Supplementary material for: Anticancer activity of Zingiber ottensii essential oil and its nanoformulations
Source: PLoS One. 2022 Jan 24;17(1):e0262335. doi: 10.1371/journal.pone.0262335 (PMC8786151; doi:10.1371/journal.pone.0262335)
Supplement: S10 Table — (PDF) [file pone.0262335.s011.pdf]

S10 Table. Cytotoxicity of ZOEO loaded nanoformulations against HeLa cells.

**Nanoformulations**	**IC_{50} value (ng of essential oil/mL)**				
	1	2	3	Mean	SD
NE-ZO-S	4.16	8.54	4.74	5.81	2.38
NE-ZO-B	>50	>50	>50	>50	-
ME-ZO-S	4.67	9.65	7.41	7.24	2.49
ME-ZO-B	>50	>50	>50	>50	-
NG-ZO-S	8.49	7.14	11.01	8.88	1.97
NG-ZO-B	>50	>50	>50	>50	-
MG-ZO-S	8.75	10.52	13.75	11.01	2.54
MG-ZO-B	>50	>50	>50	>50	-
