# Supplementary material for: Anticancer activity of Zingiber ottensii essential oil and its nanoformulations
Source: PLoS One. 2022 Jan 24;17(1):e0262335. doi: 10.1371/journal.pone.0262335 (PMC8786151; doi:10.1371/journal.pone.0262335)
Supplement: S11 Table — (PDF) [file pone.0262335.s012.pdf]

**S11 Table. Cytotoxicity of ZOEO loaded nanoformulations against K562 cells.**

| Nanoformulations | $IC_{50}$ value (ng of essential oil/mL) | | | | |
|---|---|---|---|---|---|
| | 1 | 2 | 3 | Mean | SD |
| NE-ZO-S | 31.18 | 33.57 | 32.70 | 32.48 | 1.21 |
| NE-ZO-B | >50 | >50 | >50 | >50 | - |
| ME-ZO-S | 30.61 | 34.26 | 35.06 | 33.31 | 2.37 |
| ME-ZO-B | >50 | >50 | >50 | >50 | - |
| NG-ZO-S | 34.18 | 37.33 | 34.54 | 35.35 | 1.72 |
| NG-ZO-B | >50 | >50 | >50 | >50 | - |
| MG-ZO-S | 30.36 | 34.69 | 34.63 | 33.23 | 2.48 |
| MG-ZO-B | >50 | >50 | >50 | >50 | - |
